# Discovery and Characterization of Antiferromagnetic UFe_5_As_3_

**DOI:** 10.1021/acs.inorgchem.3c03837

**Published:** 2024-02-26

**Authors:** N. Zaremba, M. Krnel, Yu. Prots, M. König, L. Akselrud, Yu. Grin, E. Svanidze

**Affiliations:** † Max-Planck-Institut für Chemische Physik fester Stoffe, Nöthnitzer Straße 40, Dresden 01187, Germany; ‡ Ivan Franko Lviv National University, Kyryla i Mefodia St. 6, 29005 Lviv, Ukraine

## Abstract

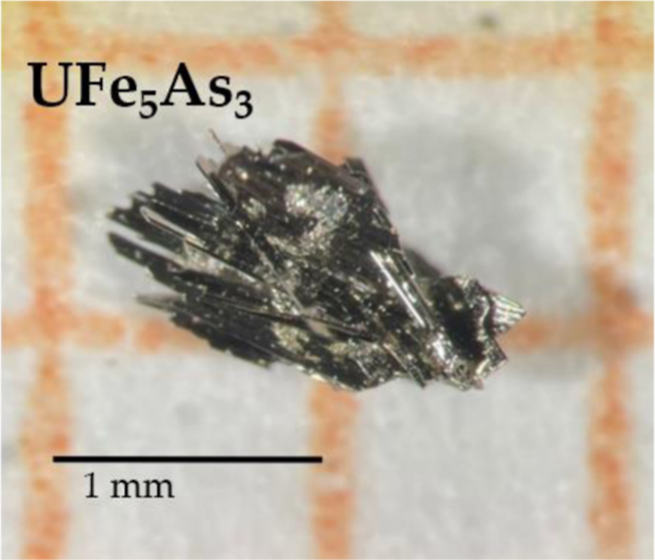

This work presents a study on a new uranium iron arsenide UFe_5_As_3_. By implementing Bi-flux synthesis, we were able to grow mm-sized single crystals of this compound, which show twinning. UFe_5_As_3_ is one of only two known uranium iron arsenides. It adopts a monoclinic, UCr_5_P_3_-type crystal structure (space group *P*2_1_/*m*, Pearson symbol *mP*18, *a* = 7.050(2) Å, *b* = 3.8582(9) Å, *c* = 9.634(1) Å, β = 100.25(1)°). The magnetic susceptibility of UFe_5_As_3_ indicates it to be an antiferromagnet with *T*_N_ = 47 K and μ_eff_ = 4.94 μ_B_ per formula unit, signaling that both U and Fe are likely magnetic in this material. The material appears to be anisotropic, with a small (likely ferromagnetic) spin reorientation transition around *T* = 29 K. The Sommerfeld coefficient γ_0_ = 135 mJ mol^–1^ K^–2^ suggests enhanced effective electron mass in UFe_5_As_3_, while electrical resistivity indicates metallic, Kondo-like behavior.

## Abstract 2

A new uranium iron arsenide is grown in single-crystalline form. Consequent characterization shows that the monoclinic structure of UFe_5_As_3_ can be represented by an arrangement of uranium-centered “shamrocks”, which form linear chains. An antiferromagnetic ordering below 47 K is observed, with both uranium and iron likely contributing to the magnetism of UFe_5_As_3_. An enhancement of the effective electron mass is suggested by a moderate Sommerfeld coefficient.

## 1 Introduction

As new classes of superconducting materials emerge, puzzles of high-temperature superconductivity continue to be one of the pressing issues in condensed matter physics and solid-state chemistry. In particular, iron–arsenic superconductors still pose many open questions.^1,2^ Given the chemical similarities between f-elements and alkali/alkali earths, we can stipulate the formation of f-electron-based compounds with the architecture of other ternary iron arsenides. Also, while the f-element-based iron–arsenic compounds are not likely to be high-*T*_c_ superconductors, we can observe what happens when more electronically complex f-electrons are placed inside crystal structures that show unconventional behavior^2−4^ and use their small energy scales for easy tuning from one ground state to another. Indeed, it has been noted previously that the chemistry of f-elements with arsenic is quite diverse,^5,6^ with several lanthanide-iron-arsenic ternaries examined during the search for new compounds with peculiar chemical and physical features. Surprisingly, only a handful of stoichiometries have been discovered so far: RFeAs (R = La, Ce, Pr, and Nd^7^), RFe_2_As_2_ (R = Eu^8^), R_12_Fe_57.5_As_41_ (R = La and Ce^9^), RFe_4_As_12_ (R = Ce, Pr, Nd, Sm, Gd, Tb^10−15^), and R_6_Fe_13_As (R = Pr and Nd^16^).

Going from 4f to 5f elements, even more structural diversity may be expected.^17−21^ However, very few actinide-based iron–arsenic materials have been reported so far, perhaps as a result of synthesis complications imposed by toxicity, reactivity, and high vapor pressure of constituent elements. Prior to the current work, only one compound has been reported to exist in the uranium–iron–arsenic^22^ and thorium–iron–arsenic^10,23^ systems. The arsenide UFeAs_2_ (HfCuSi_2_ structure type, space group *P*4/*nmm*) was studied several decades ago, with measurements of physical properties impeded by the presence of impurities.^22^ A skutterudite material ThFe_4_As_12_^10,23^ was examined due to its unusual enhancement of the Sommerfeld coefficient, which remains unexplained.

In this work, we successfully revisit the uranium–iron–arsenic ternary phase diagram and report the discovery and characterization of the new arsenide UFe_5_As_3_ in single-crystalline form. This compound crystallizes in a monoclinic space group, which can be represented by a chain of interconnected “shamrocks”. Among compounds with the same 1:5:3 stoichiometry, the “shamrock” description can be applied to LaCo_5_P_3_ (SrNi_5_P_3_-type structure^24^), YNi_5_Si_3_ (YNi_5_Si_3_-type structure^25^), YCo_5_P_3_ (YCo_5_P_3_-type structure^26^), and UCo_5_Si_3_ (UCo_5_Si_3_-type structure^27^). It is likely that the arrangement of the “shamrocks”, i.e., straight vs zigzag chains vs islands, dictates the particular magnetic behavior; however, for most of the aforementioned materials, magnetic properties remain unknown.

## 2 Materials and Methods

**Figure 1:**
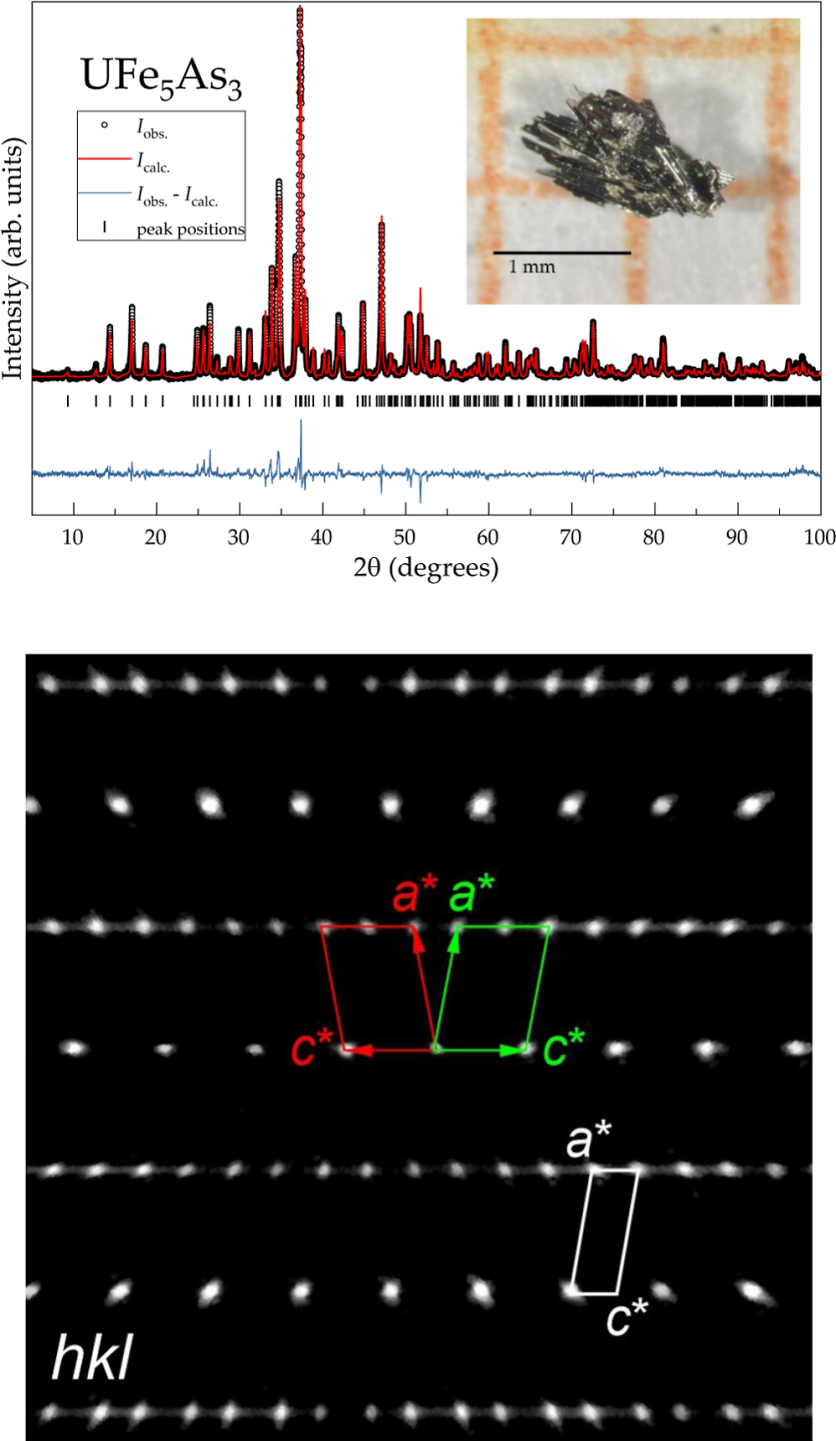
Top panel: powder XRD pattern obtained from powdered single crystals of UFe_5_As_3_ (*I*_obs._, symbols), together with the calculated profile (*I*_calc._, red line), difference between them (*I*_obs._ – *I*_calc._, blue line), and calculated positions of the Bragg reflections (vertical ticks). Inset: agglomeration of the needle-like single crystals of UFe_5_As_3_. Bottom panel: a reconstructed image of the diffraction pattern of UFe_5_As_3_ along [010]* generated from the collected single crystal data, with a schematic representation of the twin component contributions (red and green cells). The white parallelogram indicates a unit cell obtained by automatic indexing of the collected data set.

All sample preparation and handling was performed in the specialized laboratory, equipped with an argon-filled glovebox system (MBraun, p(H_2_O/O_2_)< 0.1 ppm).^28^ Single crystals of UFe_5_As_3_ were grown from the Bi flux. Pure elements of uranium (sheets, Goodfellow, 99.98%), iron (powder, ChemPur, 99.9%), arsenic (pieces, Puratronic, 99.999%), and bismuth (granules, ChemPur, 99.999%) in the ratio 1:5:3:20 were placed in an alumina crucible and subsequently sealed in a tantalum tube. The tube was heated in a vertical tube furnace to 1150 °C for 24 h, held at this temperature for 12 h, and slowly cooled to 700 °C for 240 h with further cooling to room temperature for 96 h in a vertical furnace. After the reaction, the tantalum tube with the mixture was then sealed in the silica tube, heated to 500 °C, and placed into the centrifuge to separate Bi flux from the sample. The resultant product consisted of shiny gray needle-shaped crystals of UFe_5_As_3_, which typically grow in clusters (inset of Figure 1, top panel). Crystals appear to be stable in air and have residual Bi on the surface (<2%), which does not affect their properties. It was not, however, possible to synthesize the ThFe_5_As_3_, YFe_5_As_3_, or LuFe_5_As_3_ compounds to be used as nonmagnetic analogues of UFe_5_As_3_. While the composition of La_12_Fe_57.5_As_41_ is close to 1:5:3, it crystallizes in a different structure type.^9^

Powder X-ray diffraction was performed on a Huber G670 image plate Guinier camera with a Ge-monochromator (CuK_α1_ radiation, λ = 1.54056 Å), Figure 1 (top panel). Phase identification was done using the WinXPow software.^29^ The lattice parameters were determined by a least-squares refinement using the peak positions, extracted by profile fitting (WinCSD software^30^). For single-crystal experiments, relatively thin (∼20 μm) but long (∼200 μm) specimens were used (see, for example, inset of Figure 1). The diffraction data were collected using a Rigaku AFC7 diffractometer equipped with a Saturn 724+ CCD detector and MoK_α_ radiation (λ = 0.71073 Å). The WinCSD^30^ and SHELXL^31^ software packages were used for structure solution and refinement data analysis. The complete crystallographic information is given in Table 1.

**Table 1:** Crystallographic Data of UFe_5_As_3_

composition	UFe_5_As_3_
structure type	UCr_5_P_3_
space group	*P*2_1_/*m* (monoclinic)
*Z*	2
Pearson symbol	*mP*18
lattice parameters^a^	
*a*/Å	7.050(2)
*b*/Å	3.8582(9)
*c*/Å	9.634(1)
*V*/Å^3^	257.87(9)
β/°	100.25(1)
calc. density/g cm^–^^1^	9.56
range in *h*, *k*, *l*	–10 ≤ *h* ≤ 10
	–5 ≤ *k* ≤ 5
	–14 ≤ *h* ≤ 14
absorption coeff./mm^–^^1^	64.0
*N*(*hkl*) measured^b^	1568
*N*(*hkl*) observed	1474
refined parameters	60
twin ratio	0.544(2):0.456
*R*1	0.0329
*wR*2	0.0862
residual peaks/e Å^–^^3^	–5.36/4.22

a From powder diffraction data.

b Total number of reflections in the *hklf*5 file (common and individual reflections of each component), not averaged due to the twinning.

The single crystals of UFe_5_As_3_ were additionally analyzed by energy-dispersive X-ray spectroscopy with a Jeol JSM 6610 scanning electron microscope equipped with an UltraDry EDS detector (Thermo Fisher NSS7). The semiquantitative analysis was performed with a 30 keV acceleration voltage. No impurity elements were observed, confirming that no reaction with the crucible took place during synthesis. The experimentally determined element ratio of U:Fe:As was 9(2):59(2):32(2), which is in good agreement with the 9.9:58.7:31.4 composition obtained from the structure solution.

Temperature- and field-dependent magnetic measurements were conducted in a Quantum Design Magnetic Properties Measurement System (MPMS-XL). Several single crystals of UFe_5_As_3_ were mounted on a quartz capillary. The magnetic moment was measured at temperatures ranging from 2 to 600 K and in magnetic fields up to *H* = 7 T (applied along and perpendicular to the [010] axis of the crystals). The specific heat data were collected on a QD Physical Property Measurement System (PPMS) from *T* = 0.4 K to *T* = 100 K in *H* = 0 and *H* = 9 T magnetic fields. For measurements of electrical resistivity, a microscale device was fabricated out of a UFe_5_As_3_ single crystal by using a plasma focused ion beam (FIB).^32^ AC electrical resistivity measurements were performed by a QD PPMS using a standard four-probe technique at temperatures between *T* = 2 and 300 K in *H* = 0 and *H* = 9 T applied magnetic fields. A current pulse of 0.01 mA with a frequency of 93 Hz for 1 s was applied along the [010] axis of the UFe_5_As_3_ crystal.

## 3 Crystal Structure Determination

**Figure 2:**
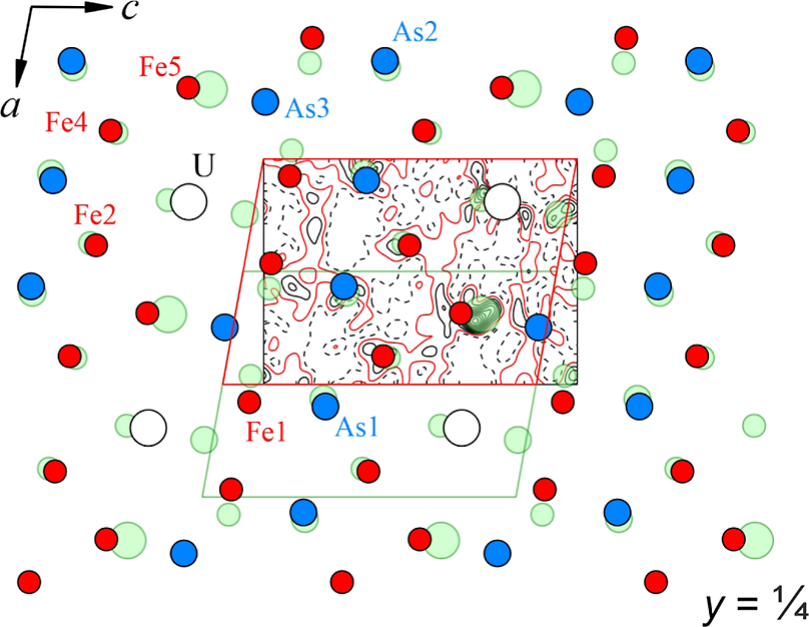
Difference density map in the (040), i.e., *x*^1^/_4_*z*, plane in UFe_5_As_3_ (black: isolines with a step of 1 e Å^–3^ and red: zero lines). The atomic positions of the original UCr_5_P_3_-type structure are shown in color (U: white, Fe: red, and As: blue). The additional maxima can be interpreted with the second UCr_5_P_3_-type motif shifted along [100] by ca. ^1^/_2_ (transparent green circles).

An indexation of the collected diffraction data set resulted in the monoclinic unit cell with the lattice parameters: *a* = 7.05 Å, *b* = 3.86 Å, *c* = 19.26 Å, and β = 100.3°. Nevertheless, a careful examination of the data clearly indicates that the observed extinction conditions are not compatible with any known space group (Figure 1). More precisely, for *hkl* reflections with *h* = 2*n*, only every second reflection is present. Such a picture is typical for the formation of a twinned agglomerate. Note that twinning is also observed for the isotypic UCr_5_P_3_ compound.^33^ The observed reflections can therefore be assigned to two domains (*a* = 7.05 Å, *b* = 3.86 Å, *c* = 9.63 Å, and β = 100.3°). Initial data reduction was performed for each domain separately. The analysis of the individual reflections *hkl* with *h* = 2*n* + 1 of each component delivered the estimated ratio of the twin components of 0.57:0.43. The reflections of the majority component were used for the crystal structure solution. For this purpose, the intensities of the common reflections (*h* = 2*n*) were roughly scaled by a factor of 0.57 (the fraction of this component in the twinned agglomerate). Crystal structure solution was performed in the space group *P*2_1_/*m* by direct methods, which delivered a structure model with 1U, 5Fe, and 3As atomic sites. The refinement resulted in acceptable residuals *R*1 = 0.0665 and *wR*2 = 0.1648. In the next step, the collected images were processed using both domains and applying a twinning matrix (−1 0 0 0–1 0 0.5 0 1). The obtained *hklf*5 data set was used for the final runs. The refinement resulted in the residuals *R*1 = 0.0371 and *wR*2 = 0.0936 and a twin component ratio of 0.544(2):0.456. The latter is very close to those obtained by estimating the intensities of the individual reflections in the initial steps of the crystal structure determination. From the difference Fourier map, first, an additional maximum was observed (12.19 e Å^–2^) close to the Fe5 position (0.64 Å). Nevertheless, independent refinement of the occupancy parameters for both split positions assuming occupation by iron reduced further residuals (*R*1 = 0.0332, *wR*2 = 0.0880) revealed a practically negligible defect at the Fe5 site of 0.979(5), which is equal to one within 3 esd, but strong overoccupancy of the second split position of 0.072(6), resulting in a nonphysical total occupation of 1.04 for both positions. A subsequent attempt to occupy the second split site by As did not change the residuals but slightly reduced the total occupancy for both split sites to 1.032. Finally, the occupancy of the second split site by uranium resulted in a total occupancy of 1.00 (Fe5:0.982(5), U2:0.018(2)), which (within 2 esd) would satisfy the condition that both positions exclude each other within the same structural matrix. However, the distances of U2 to its neighbors are either completely nonphysical (*d*_U2–As3_ = 1.76 Å) or much too short in comparison with other closest contacts for U1 (2.57 Å for Fe1, 2.70 Å for Fe3, and 2.76 Å for As1). These findings indicate that the appearance of the split position is not caused by structural problems within the original UCr_5_P_3_-type matrix in UFe_5_As_3_ but rather signals the experimental difficulties with the separation of reflections of different twin domains or the presence of local structural modifications (extended defects or inclusions of other structural segments similar to them were recently found in Be_2_Ru^34^), leading to the partial violation of translational symmetry. The first scenario was evaluated by inclusion into the refinement of weak high-angle reflections, which are well separated in reciprocal space and can be easily integrated separately. This operation increased the residuals but did not suppress the appearance of the split sites. The scenario with the local violation of translational symmetry is in line with the twinning already presented in the crystals investigated. Indeed, the part of the maxima in the difference density map can be interpreted by the presence of the minor amount of related structural motif but shifted along [100] by approximately ^1^/_2_ (Figure 2). This would imitate a local YNi_5_Si_3_-type structure arrangement (Figure 4). However, this assumption does not describe the difference density map completely, indicating the presence of more sophisticated deviations from the translational symmetry. The refinement of such a model yields two motifs in a ratio of 0.98 to 0.02 and further reduces the residuals (*R*1 = 0.0331, *wR*2 = 0.0867). All these evaluations allow the conclusion that the crystal structure of UFe_5_As_3_ is representative of the structure type UCr_5_P_3_ with ordered occupancy of all positions. Due to the applied crystal growth technique from the Bi melt and the monoclinic symmetry, the experimentally obtained single crystals tend to form extended defects—such as twinning—which may affect the resultant physical properties. The unusual occupancy of the split positions indicates not the problems in the original UCr_5_P_3_-type structure matrix but rather signals the presence of local structural features, leading to the partial violation of translational symmetry, which is in line with the twinning already present in the investigated crystals. Thus, for the final runs, a completely ordered model of UCr_5_P_3_ was applied. The presence of an additional peak (in the difference Fourier map) was be attributed to an additional structural motif arising from a minority phase in the examined specimen (∼2%). The values of interatomic distances are given in Table S1, while the final atomic coordinates and displacement parameters are listed in Table S2.

**Figure 3:**
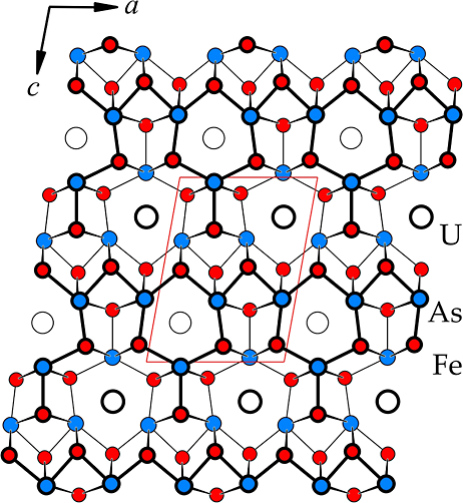
Crystal structure of UFe_5_As_3_ represented as a network of interconnected Fe and As atoms (*d*_Fe–As_ = 2.35–2.61 Å).

In the crystal structure of UFe_5_As_3_ (Figure 3), all atoms are located on two mirror planes at *y* = ^1^/_4_ and ^3^/_4_. In a large family of compounds with a metal to metalloid ratio of 2:1,^24−27,35−45^ atoms form a tessellation of slightly distorted hexagonal [Fe_6_As_6_], pentagonal [Fe_6_As_4_], tetragonal [U_2_Fe_4_As_2_], and trigonal [U_2_Fe_4_] prisms.

An analysis of interatomic distances in UFe_5_As_3_ (Table Supporting Information) reveals Fe–As distances (marked in yellow in Table S1) to be close to the sum of the covalent radii of these elements (*r*_Fe+As_ = 2.37 Å). As a result of the higher electronegativity of As (2.18 by Pauling) and the reduction of distances between Fe–As atoms, the Fe and As atoms may be considered to form a complex polyanionic framework (Figure 3). The U–Fe and U–As distances show significantly higher values compared to the sum of covalent radii (*r*_U_ + *r*_Fe_ = 2.58 Å and *r*_U_ + *r*_As_ = 2.63 Å). The uranium–uranium shortest contact *d*_(U–U)_ = 3.8582(9) Å (see Table S1) is rather large (for example, in elemental uranium *d*_(U–U)_ = 2.75–3.43 Å). According to the Hill limit,^46^ such a large separation of the uranium atoms is likely to yield a magnetic ground state, as described in Section 4.

**Figure 4:**
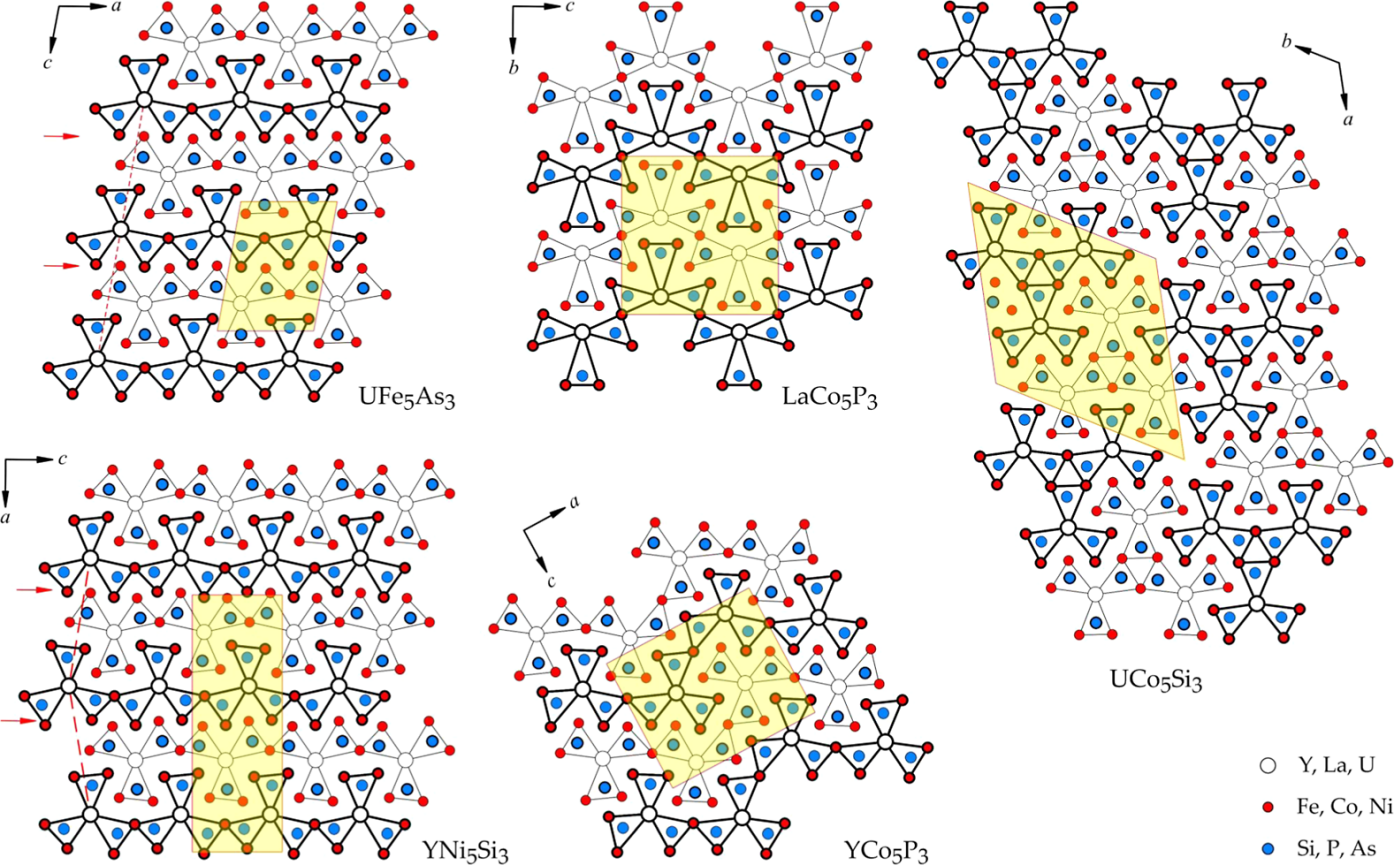
Crystal structures of UFe_5_As_3_, LaCo_5_P_3_, UCo_5_Si_3_, YNi_5_Si_3_, and YCo_5_P_3_ represented as condensed trigonal prisms around nonmetal atoms (As, Si, or P). All atoms are located on two parallel planes, highlighted by thin and thick lines, respectively. Unit cells for each of the structures are highlighted in yellow. Red dashed lines in the structures of UFe_5_As_3_ and YNi_5_Si_3_ emphasize the relative arrangement of the similar building blocks (linear “shamrock” chains). The red arrows indicate similar fragments of both structures. These are likely responsible for the twinning during the crystal growth and the local violation of the translational symmetry.

Within the structure of UFe_5_As_3_, the trigonal prisms [U_2_Fe_4_] filled with a metalloid atom (As) can be identified. Their condensation through the U−U edge creates so-called “shamrock” segments, which share the Fe−Fe edges, forming endless chains along [100] (Figure 4). The composition of “shamrock” chains in UFe_5_As_3_ can be written as UFe_4_Fe_2/2_As_3_, where Fe_2/2_ are two atoms which are shared between the “shamrock” segments in such a chain. The compounds with similar 1:5:3 stoichiometry can all be described as patterns formed by “shamrocks”, as shown in Figure 4. For example, both UFe_5_As_3_ (UCr_5_P_3_-type structure) and YNi_5_Si_3_ (YNi_5_Si_3_-type structure^25^) form similar, straight “shamrock” chains. These chains have different arrangements in the direction perpendicular to the chain. Other compounds form either zigzag “shamrock” chains—LaCo_5_P_3_ (SrNi_5_P_3_-type structure^24^) and YCo_5_P_3_ (YCo_5_P_3_-type structure^26^)—or isolated islands of “shamrock” triangles, as is the case for UCo_5_Si_3_ (UCo_5_Si_3_-type structure^27^).

## 4 Magnetic Properties

**Figure 5:**
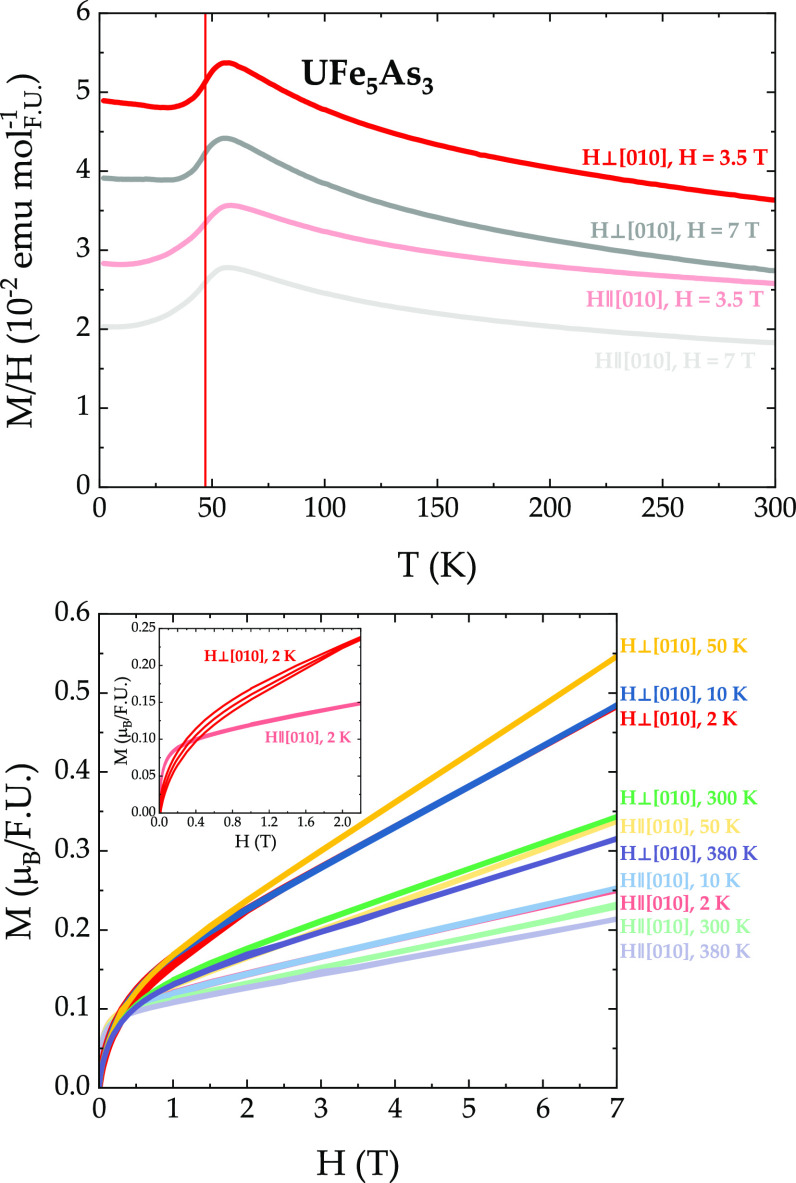
Top panel: magnetic susceptibility of UFe_5_As_3_ in *H* = 3.5 T (pink) and *H* = 7 T (gray) applied magnetic fields. Bottom: magnetic isotherms, taken at temperatures below (red and blue) and above (yellow, green, and purple) *T*_N_ = 47 K. *H*⊥[010] and *H*∥[010] are shown in darker and lighter colors, respectively.

**Figure 6:**
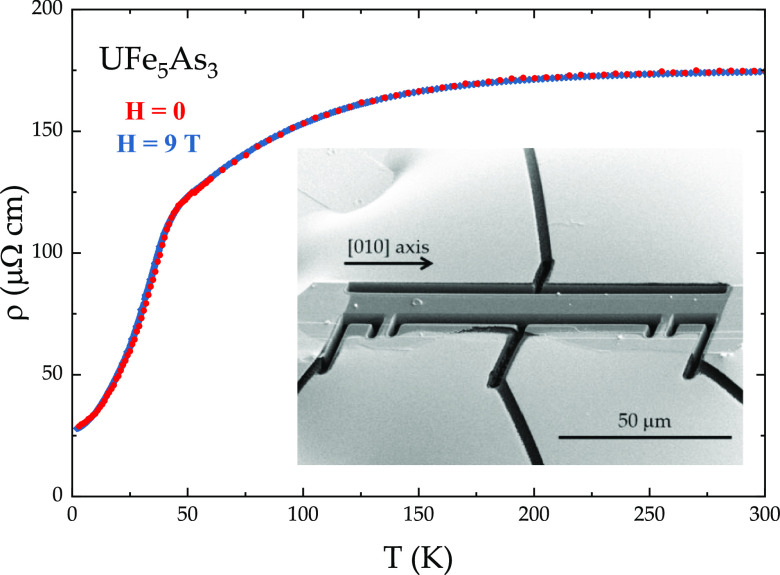
Electrical resistivity of UFe_5_As_3_ along [010] measured in *H* = 0 (red) and *H* = 9 T (blue) magnetic fields. Inset: a microscale device made from a UFe_5_As_3_ single crystal with the help of FIB. The electric current *i* was applied along the [010] axis of the crystal (running horizontally).

The temperature-dependent magnetic susceptibility *M*/*H* (Figure 5, top panel) reveals an antiferromagnetic ordering below *T*_N_ = 48.5 K for both directions of the external magnetic field (see d*MT*/d*T*, shown in Figure S1, top panel). The position of the feature associated with entrance into the ordered state appears to be isotropic and is not affected by the magnitude of the applied magnetic field. The difference in magnetic susceptibility for *H* = 3.5 T (pink) and *H* = 7 T (gray) magnetic fields can possibly be attributed to the presence of a small (ppm) amount of ferromagnetic impurities, such as, for example, elemental iron. The overall amount of such a magnetic impurity must be below 1 at. %, given that it was not possible to detect it by means of powder X-ray diffraction or energy-dispersive X-ray spectroscopy. The effective magnetic moment μ_eff_ was estimated from the inverse susceptibility (Figure 5, top panel, right axis), which was fit with the Curie–Weiss law for temperatures above *T* = 60 K. The resultant effective moments are μ_eff_ = 4.76 μ_B_ (*H*⊥[010]) and μ_eff_ = 4.72 μ_B_ (*H*∥[010]) per formula unit. A comparison with the range for the theoretical values of μ_eff_ for U (μ_eff,theory_ = 3.43–3.62 μ_B_) and Fe (μ_eff,theory_ = 1.73–4.90 μ_B_) suggests that both elements are likely contributing to the magnetism of UFe_5_As_3_. The values of the Weiss temperature θ_W_ = −57 K (*H*⊥[010]) and θ_W_ = −110 K (*H*∥[010]) are rather different, reflecting the anisotropy of this material. It is possible that the larger θ_W_ signals Kondo lattice hybridization (which is also consistent with the character of resistivity, see Figure 6), while the smaller θ_W_ is on the order of the Neel temperature *T*_N_ = 47 K. Another scenario behind the difference between the values of θ_W_ along two different directions can be magnetic frustration, which is possible given the triangular arrangement of uranium (and iron) atoms within the *ac*-plane (see Figure 3).

The magnetic isotherms, taken at various temperatures, are shown in the bottom panel of Figure 5. A clear anisotropy is evident when we compare the data for *H*⊥[010] (bright colors) and *H*∥[010] (pastel colors). A small upturn at low fields is observed for the data taken above and below the ordering temperature *T*_N_ = 47 K, which is consistent with the presence of a small amount of ferromagnetic impurities such as elemental iron. For the *T* = 2 K curve, a small hysteresis is observed when the magnetic field is applied perpendicular to the [010] axis. This suggests that perhaps a small ferromagnetic component within the *ac*-plane exists in UFe_5_As_3_. Another indication of this comes from a secondary transition observed in specific heat and resistivity around *T* = 29 K, which will be discussed below.

Due to the relatively small thickness and mechanical fragility of the UFe_5_As_3_ single crystals, FIB microscale structuring was applied in order to study the electrical resistivity of this compound. An example of a microscale device is shown in the inset of Figure 6—the current is applied along the [010] axis of the crystal. The temperature dependence of the electrical resistivity along [010] indicates metallic behavior for the whole temperature range. An entrance into the ordered state is marked by a feature around *T* = 36 K (see dρ/d*T*, shown in Figure S1). It is important to note that the value of the ordering temperature appears to be nearly 10 K lower compared to those extracted from specific heat and magnetization data. Since the resistivity measurements were taken on a microscale device, it is possible that there is some non-negligible strain induced by the mounting of the crystal, which resulted in a decrease of the ordering temperature. This suggests that perhaps pressure or strain investigations of UFe_5_As_3_ can be used to suppress the magnetic order of this system. As is the case for the magnetic susceptibility data, the application of a magnetic field does not affect the ordering temperature. The relatively low residual resistivity ratio (RRR) of 6 is in agreement with the local violation of translational symmetry (see Section 3). In the derivative of the resistivity data, another transition is observed around *T* = 29 K, the origin of which will be discussed below. The overall shape of the electrical resistivity of UFe_5_As_3_ is consistent with the presence of Kondo lattice hybridization in this material. This is further confirmed by a fairly large Sommerfeld coefficient (γ_0_ = 135 mJ mol^–1^ K^–2^) as well as a large and negative value of the Weiss temperature (θ_W_ = −110 K for *H*∥[010]).

**Figure 7:**
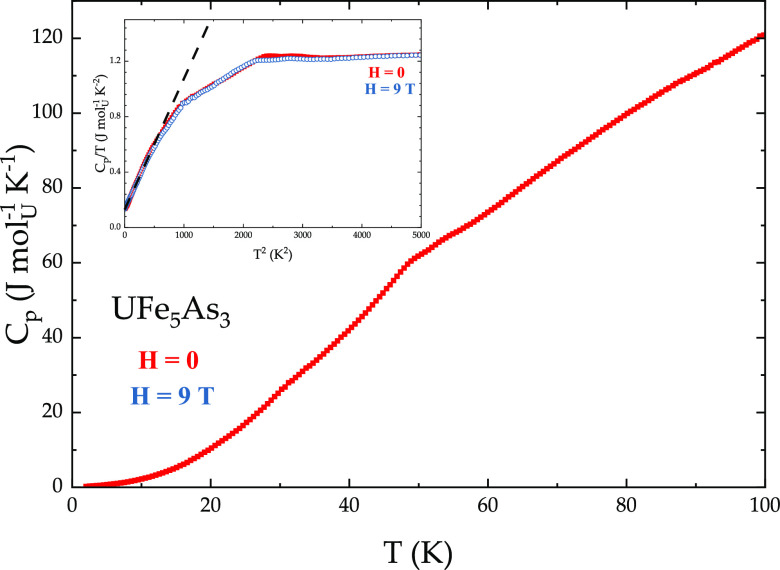
Temperature-dependent specific heat of UFe_5_As_3_ in *H* = 0 (red) and *H* = 9 T (blue). Inset: the *C*_p_/*T* vs *T*^2^ data in *H* = 0 (red) and *H* = 9 T (blue) with the dashed line representing the linear fit from which the values of γ_0_ and β were extracted.

Similar to the resistivity data, in the specific heat measurements (Figure 7), two transitions are observed—one at *T* = 46 K and another at *T* = 29 K. Both transitions do not appear to be affected by an application of the magnetic field. The lower transition can likely be associated with a spin reorientation. Another possibility is that one of the magnetic transitions corresponds to the ordering of the iron sublattice, while the other one marks the ordering of the uranium one, with further details hopefully achievable by future magnetic studies of UFe_5_As_3_. From the *C*_p_/*T* vs *T*^2^ plot (Figure 7, inset), the value of the Sommerfeld coefficient γ was extracted by fitting the data with the Debye model. The fit (dashed line) yields γ_0_ = 135 mJ mol_U_^–1^ K^–2^ and β = 0.92 mJ mol_U_^–1^ K^–4^ (θ_D_ = 267 K). Since the synthesis of the nonmagnetic analogue ThFe_5_As_3_ was unfortunately not successful, it was not possible to estimate the magnetic contribution to the specific heat of UFe_5_As_3_ (this also means that the analysis of entropy associated with magnetic ordering in this compound was not carried out). It is therefore feasible that the value of γ_0_, extracted from the Fermi liquid fit at low temperatures, is an overestimate. Assuming that the relatively large value of γ_0_ is in fact accurate, this may indicate the heavy-fermion character of UFe_5_As_3_, albeit with a modest effective mass enhancement. This is further supported by the Kondo-like resistivity of UFe_5_As_3_ (see Figure 6). In our previous work, we postulated a set of empirical ingredients that are likely to yield effective mass enhancement in uranium-based materials—the mass percentage of uranium below 40%, the coordination number of uranium above 12, and the shortest uranium contact above 3 Å.^47^ In UFe_5_As_3_, effective mass is only slightly enhanced, which could perhaps be explained by the fact that only two of the three requirements are fulfilled: the mass percentage of uranium is 32%, the coordination of uranium is 18, and the shortest uranium distance is 2.93 Å. The final value of the ordering temperature *T*_N_ = 47 K for UFe_5_As_3_ was established as an average between the values of the maximum in (i) d*MT*/d*T* (48.5 K, Figure S1, top panel), (ii) dρ/d*T* (36 K, Figure S1, middle panel), and (iii) d*C*_p_/d*T* (46 K, Figure S1, bottom panel). It is likely that both uranium and iron sites have small magnetic moments, similar to what has been reported for UFe_2_ (*T*_C_ = 165 K^48−51^), UFe_5_Si_3_ (*T*_C_ = 310 K^52,53^), and U_2_Fe_12_Al_5_ (*T*_C_ = 295 K^54^). A more quantitative assessment regarding the respective contributions of iron and uranium to the magnetism of UFe_5_As_3_ can hopefully be obtained as part of a future study.^55,56^

## 5 Discussion and Conclusions

Among uranium-based iron arsenides, only two compounds—UFe_5_As_3_ and UFeAs_2_^22^—have been discovered so far. Surprisingly, the overall number of lanthanide iron arsenides and phosphides with the same stoichiometry is also rather scarce.^33,57−61^ In this work, the discovery and characterization of a new uranium iron arsenide UFe_5_As_3_ are presented. It was possible to grow large, mm-sized single crystals of this compound, which crystallizes in the UCr_5_P_3_-type structure and shows extended defects, including twinning. This system orders antiferromagnetically below *T*_N_ = 47 K, with features corresponding to magnetic ordering observed in magnetization, specific heat, and resistivity data. It appears that both U and Fe atoms participate in the ordering as the effective magnetic moment is estimated to be μ_eff_ = 4.94 μ_B_ per formula unit. A relatively low RRR = 6 of this metal can be explained by twinning.

It is interesting to compare UFe_5_As_3_ with UFe_5_Si_3_, which orders ferromagnetically at *T*_C_ = 310 K.^52,53^ The crystal structure of the latter compound cannot be represented in the form of “shamrock” segments, as shown in Figure 4. Nonetheless, the shortest U–U distances for two materials are virtually the same—*d*_U–U_ = 3.8582(9) Å for UFe_5_As_3_ vs *d*_U–U_ = 3.929(1) Å for UFe_5_Si_3_. The large difference in their ordering temperature can perhaps be explained by a more compact packing of the UFe_5_Si_3_ lattice, with the shortest Fe–Fe distance *d*_Fe–Fe_ = 2.439(1) Å being smaller than that of UFe_5_As_3_ (*d*_Fe–Fe_ = 2.6058(2) Å). This, in turn, seems to result in stronger Fe–Fe correlations, yielding a higher ordering temperature in the UFe_5_Si_3_ compound. It has been suggested that among the U–Fe–Si compounds, magnetism is driven predominantly by itinerant electrons.^52^ Our preliminary analysis of another newly discovered compound UFe_4_As_2_^62^ indicates that, much like in UFe_5_As_3_, the uranium 5f orbitals in this system appear to be highly delocalized.

In the UFe_5_As_3_ compound, a partial substitution on the iron site could potentially result in the formation of quaternary ordered variants of the UCr_5_P_3_-type structure, similar to what has been reported for the rare-earth phosphides, which form quaternary ordered variants of the YCo_5_P_3_-type structure.^63^ By replacing iron with a smaller or larger atom, the effects of chemical pressure can thus be examined. In particular, it appears that even modest compression of UFe_5_As_3_ (as a result of microscale device preparation, see Figure 6 and discussion therein) leads to a significant change in the ordering temperature. This suggests that negative chemical pressure (i.e., Ni or Co) might perhaps provide a route toward further suppression of *T*_N_ in this system. Of course, the effects of electron change in the case of non-isoelectronic doping should also be taken into consideration as they will certainly influence the resultant properties.

## Supporting Information Available

Crystallographic, magnetization, specific heat, and electrical resistivity data (PDF)
