# Supplementary material for: Discovery and Characterization of Antiferromagnetic UFe5As3
Source: Inorg Chem. 2024 Feb 26;63(10):4566–73. doi: 10.1021/acs.inorgchem.3c03837 (PMC10934805; doi:10.1021/acs.inorgchem.3c03837)
Supplement: Supplementary file 1 — ic3c03837_si_001.pdf [file ic3c03837_si_001.pdf]

Discovery and characterization of antiferromagnetic UFe_5As_3
Supporting Information

N. Zaremba, M. Krnel, Yu. Prots, M. König, L. Akselrud, Yu. Grin, and E. Svanidze[a]
Max-Planck-Institut für Chemische Physik fester Stoffe, Nöthnitzer Straße 40, Dresden 01187, Germany

TABLE S1. Interatomic distances (δ, Å) for UFe_5As_3.

Atom	δ, Å	Atom	δ, Å	Atom	δ, Å
U – 2As2	2.928(1)	Fe1 – 1As1	2.360(2)	As1 – 1Fe2	2.350(2)
2As1	2.931(1)	1As3	2.410(2)	1Fe1	2.360(2)
2As3	2.976(8)	2As3	2.447(1)	1Fe4	2.385(3)
2Fe1	3.068(1)	2Fe1	2.740(2)	2Fe4	2.464(1)
1Fe2	3.136(2)	1Fe3	2.746(2)	2Fe5	2.586(2)
2Fe3	3.162(1)	2Fe5	2.959(2)	2U	2.931(1)
1Fe3	3.166(2)	2U	3.068(1)	As2 – 1Fe3	2.348(2)
1Fe1	3.207(2)	1U	3.207(2)	1Fe2	2.389(2)
1Fe4	3.274(2)	Fe2 – 1As1	2.350(2)	2Fe2	2.415(1)
2Fe4	3.311(1)	1As2	2.389(2)	1Fe4	2.421(3)
1Fe5	3.529(2)	2As2	2.415(1)	2Fe5	2.609(2)
1Fe5	3.644(2)	2Fe4	2.604(2)	2U	2.928(1)
2U	3.858(1)	2Fe2	2.619(2)	As3 – 1Fe1	2.410(2)
		1Fe5	2.630(3)	1Fe5	2.430(2)
		2Fe5	2.930(2)	1Fe3	2.431(2)
		1U	3.136(2)	2Fe3	2.443(1)
		Fe3 – 1As2	2.348(2)	2Fe1	2.447(1)
		1As3	2.431(2)	2U	2.976(8)
		2As3	2.443(1)		
		2Fe3	2.656(2)		
		1Fe1	2.740(2)		
		2Fe5	3.013(2)		
		1U	3.1663(1)		
		2U	3.1664(1)		
		Fe4 – 1As1	2.385(2)		
		1As2	2.421(3)		
		2As1	2.464(1)		
		2Fe2	2.604(2)		
		2Fe4	2.647(2)		
		1Fe5	2.693(3)		
		1U	3.274(2)		
		2U	3.311(1)		
		Fe5 – 1As3	2.430(0)		
		2As1	2.586(2)		
		2As2	2.609(2)		
		1Fe2	2.630(3)		
		1Fe4	2.693(3)		
		2Fe2	2.930(2)		
		2Fe1	2.959(2)		
		2Fe3	3.013(2)		
		1U	3.529(2)		
		1U	3.644(2)		

[a] E-mail: svanidze@cpfs.mpg.de

TABLE S2. Atomic coordinates and equivalent (isotropic) displacement parameters (in Å^2) for UFe_5As_3. All atoms lie on Wyckoff sites 2e (x1/4z).

Atom	Occupancy	x/a	y/b	z/c	$U_{eq.}$
U1	1	0.1927(8)	1/4	0.7871(4)	0.0072(8)
As1	1	0.0994(2)	1/4	0.3417(1)	0.0065(3)
As2	1	0.5681(2)	1/4	0.3321(1)	0.0064(3)
As3	1	0.7467(2)	1/4	0.9738(1)	0.0074(2)
Fe1	1	0.0770(2)	1/4	0.0944(2)	0.0084(4)
Fe2	1	0.3797(4)	1/4	0.5151(1)	0.0069(3)
Fe3	1	0.4644(2)	1/4	0.0866(1)	0.0085(4)
Fe4	1	0.8707(4)	1/4	0.4967(2)	0.0075(3)
Fe5	1	0.6841(3)	1/4	0.7175(2)	0.0088(3)
U2	0.0190(2)	0.713(3)	1/4	0.7882(2)	0.008(3)

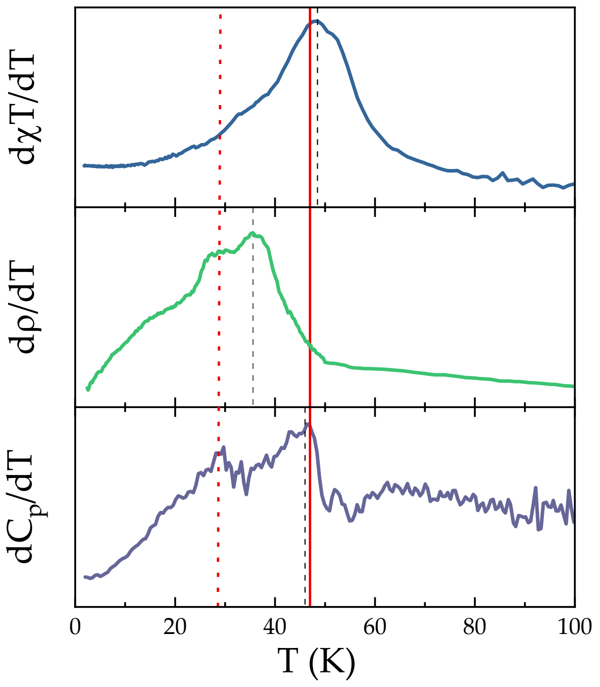

FIGURE S1. The value of the ordering temperature T_N = 47 K (solid red line) was estimated from the associated features in dMT/dT (48.5 K, top panel) and dC_p/dT (46 K, bottom panel). The feature corresponding to the entrance into magnetic state occurs at significantly lower temperature for the dρdT data (36 K, middle panel). This can perhaps be attributed to the strain, induced upon the single crystal of UFe_5As_3 as part of the micro-scale device preparation process. Dotted red line corresponds to the secondary transition observed in the specific heat data and resistivity data at T = 29 K.
